# Computational modeling of RNA 3D structure based on experimental data

**DOI:** 10.1042/BSR20180430

**Published:** 2019-02-08

**Authors:** Almudena Ponce-Salvatierra, Astha, Katarzyna Merdas, Chandran Nithin, Pritha Ghosh, Sunandan Mukherjee, Janusz M. Bujnicki

**Affiliations:** 1 Laboratory of Bioinformatics and Protein Engineering, International Institute of Molecular and Cell Biology in Warsaw, ul. Ks. Trojdena 4, Warsaw PL-02-109, Poland; 2 Bioinformatics Laboratory, Institute of Molecular Biology and Biotechnology, Faculty of Biology, Adam Mickiewicz University, ul. Umultowska 89, Poznan PL-61-614, Poland

**Keywords:** computational biochemistry, integrative modeling, molecular modeling, RNA structure

## Abstract

RNA molecules are master regulators of cells. They are involved in a variety of molecular processes: they transmit genetic information, sense cellular signals and communicate responses, and even catalyze chemical reactions. As in the case of proteins, RNA function is dictated by its structure and by its ability to adopt different conformations, which in turn is encoded in the sequence. Experimental determination of high-resolution RNA structures is both laborious and difficult, and therefore the majority of known RNAs remain structurally uncharacterized. To address this problem, predictive computational methods were developed based on the accumulated knowledge of RNA structures determined so far, the physical basis of the RNA folding, and taking into account evolutionary considerations, such as conservation of functionally important motifs. However, all theoretical methods suffer from various limitations, and they are generally unable to accurately predict structures for RNA sequences longer than 100-nt residues unless aided by additional experimental data. In this article, we review experimental methods that can generate data usable by computational methods, as well as computational approaches for RNA structure prediction that can utilize data from experimental analyses. We outline methods and data types that can be potentially useful for RNA 3D structure modeling but are not commonly used by the existing software, suggesting directions for future development.

## Introduction

RNA molecules play fundamental roles in many biological processes, such as regulation of gene expression [1–4], RNA splicing [5–7], and protein synthesis [8–10]. The understanding of these processes improves as new structures of RNA molecules and their complexes with various molecular partners are determined, and the molecular details of interactions analyzed [11–17].

As of 20 September 2018, 1345 RNA structures (and 2284 RNA complexes with other molecules) were available in the Protein Data Bank (PDB) [18], including 807 (1680) solved by X-ray crystallography, 504 (120) by NMR spectroscopy, 29 (478) by cryo-EM, and 5 (6) by other methods. However, the experimental determination of RNA structures at high resolution, which allows the description of individual atomic features as well as atomic interactions, is time-consuming, and in many cases not possible. As a result, complete high-resolution structures are available for an extremely small fraction of all the known RNA molecules that are crucial for numerous fundamental cellular processes; for instance, only 87 out of 2791 RNA families in the Rfam database have at least one experimentally determined structure.

It will be very challenging to obtain a comprehensive overview of structures for all RNA molecules that have a biochemical function (beyond protein-coding). Such efforts have been undertaken toward elucidating protein structures, e.g., through the Protein Structural Genomics initiative and related efforts around the world [19,20]. The flexible nature of biomacromolecules has proven to be a challenge to overcome when it comes to sample preparation for biophysical techniques that have a scope for their structural determination [21–23]. For instance, in crystallography, it is unusual to have a sample that readily forms well-diffracting crystals. Even the production of the sample *per se* is difficult, and biomacromolecules (proteins and nucleic acids likewise) often require some extent of engineering or chemical modification so that their structure can be analyzed with a given method [24–26]. In any of the methods used for structure determination of biomacromolecules the homogeneity of the sample is also not to be taken for granted and it poses additional difficulties as the number of different molecules involved in the formation of the macromolecular complex increases [23,27]. Furthermore, RNA molecules are known to be intrinsically dynamic, more than protein molecules, so even if a high-resolution structure of an RNA molecule is obtained, it may not represent the full spectrum of native solution conformations [28–30]. Thus, studies on RNA sequence–structure–function relationships present major challenges for macromolecular structural biology, requiring alternate approaches to gain structural information of macromolecular complexes.

Given the scarcity of experimentally determined high-resolution structures of RNA molecules, theoretical models of RNA 3D structure can assist in understanding and guiding the identification of functionally important regions and can provide a starting point for higher resolution descriptions [31]. However, ‘purely theoretical’ structural predictions usually suffer from limited accuracy, and moreover, the deviation between the theoretical model and the ‘true’ (unknown) structure is very difficult to assess in the absence of additional data. Fortunately, even though complete high-resolution structures of RNA molecules are scarce, often a wide range of heterogeneous information from biochemical and biophysical data is available. There exists various approaches for enzymatic or chemical probing of RNA secondary structure [32,33] and ways to obtain information about the overall shape of the molecule [34]. This low-resolution information can be supplemented with partial high-resolution data, e.g., structures determined for fragments of the system under study. Computational techniques can be used to integrate the existing data, guide the structure elucidation, and subsequently determine the mechanisms of action and interactions between the functional elements of the molecule [35–37]. This approach is commonly called a hybrid or integrative modeling.

This review provides necessary information about the use of experimental data that, in combination with computational approaches for the determination of RNA 3D structures, are commonly applied to study structural properties of RNA molecules. We review this issue from two complementary perspectives: experimental methods providing data that can be used in the RNA 3D structure modeling process, and computational methods that can use the experimental data. It is worth emphasizing that various experimental approaches discussed in this article have various interesting applications beyond the generation of data for macromolecular modeling: such applications are out of the scope of this review and are not discussed here. We focus primarily on the state-of-the-art and contemporary techniques while avoiding giving historical overviews. Likewise, in the description of computational methods, we concentrate on the utilization of information derived from experimental data, while ‘purely theoretical’ modeling approaches and historical perspectives are not covered. The discussion describes approaches combining experimental data (both low and high-resolution) on RNA molecules as well as their complexes, with computational modeling to obtain high-resolution models. In summary, we provide a methodological workflow to build structural models of RNA 3D structures for cases, where the use of traditional methods (X-ray crystallography, NMR, or cryo-EM) may encounter difficulties and where the application of purely theoretical modeling is unlikely to be successful.

A related important area of research, not covered by this article, is the use of computational modeling methods, in particular various types of simulations, to make predictions about various physicochemical properties of RNA molecules, which can be compared with results of experiments. The reader interested in the use of RNA modeling methods to predict functional properties of RNA molecules beyond the 3D structure is referred to recent publications on this topic, e.g., [38–40].

## Experimental methods that generate data useful for RNA 3D structure modeling

Our knowledge of RNA 2D and 3D structures is primarily based on various experimental observations. On the one hand, experiments can be interpreted in terms of specific structural knowledge about a particular RNA molecule, although large sets of experimental data collected systematically for various RNAs can be used to infer principles that apply to RNA structure in general. This review focusses on the use of experimental data to infer individual RNA structures.

### Methods that generate 3D structural information in the form of shapes

A number of experimental methods can be used to determine the overall 3D shapes of biological macromolecules. Here we review the main types of experimental approaches applicable to structural studies of RNAs alone or in complex with other molecules. We focus on the type of data generated by these methods, which can be further processed to obtain spatial restraints for building 3D structural models of RNA molecules.

#### Macromolecular X-ray crystallography

Macromolecular X-ray crystallography (MX) is the most widely used technique of structural biology because it provides highly accurate models of large macromolecular complexes [41]. In a crystallographic experiment, the crystal is exposed to an X-ray beam, and single diffraction images are collected on an area detector while rotating the crystal, which is mounted on a goniostat. The structure factors are vectorial magnitudes consisting of a module and a phase and correspond to the Fourier transform of the electron density distribution within the unit cell of the crystal. The electron density map can be calculated with the application of an inverse Fourier transform to the structure factors. While the modules can be directly obtained from the diffraction intensities measured during the experiment, the information about the phases is not recorded. The phase information has to be therefore obtained independently [42]. In a best-case scenario, a structure of a molecule related to the one under study has already been determined, and it can be used as a model for molecular replacement (MR). Roughly, the structure of the known molecule needs to be transferred into the unit cell of the unknown molecule, where rotations and translations are applied to it until a solution with the best fit between experimental diffraction data and calculated diffraction data from the model is found [43]. Alternatively, phases can be determined by Single/Multiple Isomorphous Replacement (SIR/MIR) or Single/Multiple Anomalous Dispersion (SAD/MAD) [44]. A combination of the aforementioned methods is also possible as Single/Multiple Isomorphous Replacement with Anomalous Signal (SIRAS/MIRAS) or MR with Single Anomalous Dispersion (MR-SAD). In any case, the obtained electron density map is then used to determine the 3D location of the atoms. The procedure of electron density map calculation and model building can be iterated.

One of the most significant bottlenecks of MX is the necessity to obtain well-diffracting crystals of the sample of interest. The resolution, at which crystal structures can be determined, relies highly on the size of the crystal and on how well the molecules are ordered in it. It is very difficult to obtain such crystals for RNA molecules due to their overall negatively charged surface, that often leads to non-specific interactions and causes a poor degree of internal order within the crystal [45–47].

Hydrogen atoms exhibit very low electron density; hence MX experiments cannot define their positions even when the structures are determined at very high resolution. The identification of H atoms can be addressed by neutron diffraction experiments. Although it is in principle the method of choice for the experimental determination of H atoms positions, neutron crystallography is still very challenging, e.g., the technique requires relatively large crystals, which are usually challenging to grow and there are very few facilities worldwide where the experiments can take place [48]. Despite to date no neutron structures of RNA have been determined, one potential application of this technique could be the assessment of tautomeric states of the nucleobases, which are known to play diverse roles in RNA biochemistry [49]. Currently, the positions of hydrogen atoms are rarely determined experimentally in macromolecular studies, and computational approaches are combined with chemical knowledge to predict their positions.

#### Small angle scattering

Small angle scattering (SAS) is the collective name given to various techniques, including **X-ray (SAXS)** and **neutron (SANS) scattering**, employed to characterize biological macromolecules, including RNAs. In each of these techniques, radiation is elastically scattered by a sample in solution, where molecules are randomly oriented [50]. The scattering intensity is integrated at each angle (typically 0.1–10°) to produce a 1D pattern as a function of the momentum transfer parameter [51]. In SAXS, the scattering describes the distribution of electron density that interacts with X-rays, while in SANS, it describes the distribution of the nuclei of atoms that interact with the neutrons [50,52,53]. Neutrons have two advantages over X-rays: first, they cause less molecular degradation due to radiative heating, and second, they discriminate between hydrogen and deuterium, which have opposite signs and substantial difference between the scattering lengths, which means that at certain measurable deuterium to hydrogen ratios, selected components of a complex molecular system can be made invisible [54]. In both SAXS and SANS, the scattering pattern can be analyzed to provide information about the averaged particle sizes and shapes. Depending on the angular range in which a clear scattering signal can be recorded, these techniques are capable of delivering structural information of dimensions between 1 and 100 nm [55]. The effect of averaging many randomly oriented molecules is equivalent to averaging all directions of one molecule, hence for example chirality cannot be determined [56,57]. Therefore SAXS methods require combination with other experimental approaches and/or with computational modeling to discriminate between the true structure and its mirror image.

#### Electron microscopy

Electron microscopy (EM) is a technique used for visualization of biomacromolecules, which relies on the contrast formed by the interaction of incoming electrons with the specimen. In single-particle EM, multiple 2D projection images of randomly oriented molecules are recorded, which can be then computationally combined into a 3D density map. It should be emphasized that electron micrographs are real-space images containing both amplitude and phase information. Therefore, EM techniques do not have the ‘phase problem’ that occurs in X-ray crystallography.

In negative stain EM, the sample is immobilized on the surface of a carbon film and covered with a thin layer of stain that yields high contrast images. Even though the stain limits the level of molecular detail of the reconstructed envelopes, negative stain EM has been very useful in the study of RNA assemblies, in particular when higher resolution crystal and NMR structures can be fit in the maps [58].

In cryo-EM, the sample is embedded in a thin layer of amorphous ice, which avoids the artifacts of staining. The high-energy electrons used for imaging cause, however, severe radiation damage of the individual molecules, which requires imaging with low doses of electrons. These imaging conditions yield lower contrast images, requiring the analysis of a very large number of particle images to obtain a satisfactory signal-to-noise ratio. Once a sufficient number of 2D images containing high-resolution information are captured, they are aligned and averaged into 2D classes. Particles from these classes are used to produce a 3D reconstruction. The 3D reconstruction is a Coulomb potential density map that can be interpreted similarly to electron density maps determined by X-ray crystallography. At very high resolution, cryo-EM maps can be used to determine the position of individual atoms. Currently, it is hard to say where the limits of cryo-EM are, as structures of proteins as small as hemoglobin (64 kDa) [59] have been determined, and resolutions as high as 1.8 Å have been achieved [60].

There are several examples of structures of large RNA–protein assemblies solved by cryo-EM, such as the yeast mitochondrial large ribosomal subunit [61] and the spliceosome [62]; while smaller structures, such as the Group II intron in complex with its maturase, were solved to 3.8 Å resolution [63]. Even though small RNA structures have not been determined yet with this technique alone, a combination of cryo-EM, NMR, and molecular dynamics (MD) have successfully yielded the structure of the HIV-1 RNA dimerization signal, which is only 30 kDa [64]. In comparison with other methods, cryo-EM has as a key advantage that the minimal amount of sample is required, there is no need of producing crystals and that during image classification it is possible to sort structural heterogeneity. Many 3D EM maps are however of relatively low resolution and require additional information to enable building atomic models.

#### Atomic force microscopy

Atomic force microscopy (AFM) is a type of scanning probe microscopy (SPM), which can be used for various applications, including imaging and force-probing biological samples. Unlike optical or electron microscopes that record images, AFM instruments collect data to generate images by physically touching the samples. In AFM, a molecule is immobilized on a solid substrate, such as mica or glass. Subsequently, the surface is raster scanned with sub-nanometer precision by an ultrasharp tip, mounted at the end of a force-sensing cantilever. It can be bent by repulsive and attractive interactions between the tip and the sample. The lever deflection is measured, enabling the detection of height changes with a nanometer spatial resolution, leading to a 3D topography image of the surface [65]. The tapping mode AFM, in which the cantilever is oscillated close to the sample surface without actually ‘touching’ it, found widespread application in structural biology, and in particular it has been successfully applied to image fragile biological objects, including RNA molecules [66]. Structure determination requires computational processing of AFM images. In the case of complex RNA molecules, one of the goals is to identify skeleton-like structures, from which information can be extracted about the presence of double- and single-stranded regions, their connectivity and mutual orientations [67]. Literature presents some examples of AFM application for structural characterization of RNA, including the architectural organization of the Hepatitis C virus genomic RNA [68] or the structure of the HIV-1 Rev response element (RRE) on its own and in complex with virion regulator (Rev) [69].

### Methods that generate 3D structural information in the form of spatial interactions

Another class of methods for 3D structure analysis studies interactions within (or between) molecules rather than their global shapes. These techniques offer a different point of view on the molecular structure, providing spatial restraints from which 3D molecular models can be obtained. We review the main types of experimental approaches applicable to structural studies of RNA and its complexes.

#### Nuclear magnetic resonance spectroscopy

Nuclear magnetic resonance (NMR) spectroscopy is a technique used to observe local magnetic fields around atomic nuclei and measure magnetic interactions between them. The principle behind NMR is that some nuclei isotopes (e.g., ^1^H, ^2^H, ^13^C, ^15^N, ^31^P) carry magnetic dipoles that can take up various orientations characterized by different energies when the magnetic field is applied. In response to the pulsed magnetic wave at specific (resonance) frequencies, these nuclei can absorb and re-emit electromagnetic radiation, and the obtained NMR signals are the consequence of the occurring energy state transitions [70]. The measured frequencies depend mainly on the composition of the nucleus and its chemical surrounding (i.e., covalent bonds and proximal atoms), which introduces small changes in the recorded frequencies, called chemical shifts. Magnetic interactions between nuclei connected with covalent bonds result in spin-spin coupling, while the correlation of nuclei through-space interactions can be detected by applying the nuclear Overhauser effect (NOE) [71,72]. The latter yields information about intermolecular distances of nearby nuclei and the molecular motion. Long-range structural information can be extracted from residual dipolar couplings (RDCs), which are caused by the presence of an aligning medium that interferes with the isotropic tumbling of a molecule and induces a certain degree of alignment of the molecule to the magnetic field [72]. Another important parameter is the rate at which an ensemble of nuclear spins return to the equilibrium state after the radiofrequency pulse is applied, which provides data on the atomic mobility [73].

1D NMR can also generate useful information about base pairing patterns. The imino proton resonances of G and U can only be observed when these are protected from proton exchange with the solvent. The latter happens if these protons are engaged in base pairing through hydrogen bonding interactions [74]. Counting the imino protons resonances allow the determination of the number of base pairs present in the molecule under study. 1D spectra of imino protons can inform about the type of base pairing depending on the region where the chemical shifts appear (12–15 ppm for canonical Watson–Crick base pairs, and upfield in the case of non-canonical base pairing). A combination of 1D and 2D experiments are central to the RNA secondary experimental structure determination [75,76].

After assigning recorded resonance frequencies to individual atoms in the analyzed sample, this information can be used to infer structural restraints. If the number and quality of the restraints are sufficient to define the conformations and interactions of essentially all residues in the RNA, they can be used to model its 3D structure. Traditionally it has been accomplished by energy minimization based on MD simulations that attempt to satisfy physical constraints as well as NMR-based restraints (or at least to minimize their violation) [77]. Typically, a simulated annealing protocol is used, followed by refinement, and the resulting lowest energy structures are selected to be compared with each other to check for convergence and estimate local uncertainty.

Solution NMR is an effective tool for RNA structure elucidation since its advantage relies on the collection of dynamic information of flexible macromolecules at atomistic or residue resolution. However, NMR probing of biomolecules has been limited by their size (usually <50 kDa) and so it is most successfully applied for determining the structure of small RNA domains, aptamers, and ribozymes [78–80]. NMR structure determination for larger molecules has been meeting a problem of broadening and decay of the NMR signal, making the NMR-based structural studies of large RNAs very challenging. The largest RNA molecule solved by NMR so far is the HIV-1 core packaging signal, which is 155 nucleotides long [80]. Besides, sample preparation for many types of NMR experiments requires isotope labeling of RNA molecules.

Recently developed solid-state NMR (ssNMR) is the spectroscopic method applicable to macromolecules of any size in a non-crystalline form and may become a solution to the obstacles in RNA structure determination. Although ssNMR has been rarely used in nucleic acids study and still requires more advancement and methodology preparation, recent reports suggest that it holds great promise for the study of RNAs and RNPs [81–84].

#### Electron paramagnetic resonance spectroscopy

Electron paramagnetic resonance (EPR) is a spectroscopic technique used to study chemical species with unpaired electrons. The principles behind EPR are analogous to those of NMR, with the difference in electron spins being excited instead of the spins of atomic nuclei [85]. Radicals are very reactive, and so do not normally occur in stable forms in nucleic acids or proteins. Therefore, biomacromolecules or their parts usually have to be labeled (chemically modified) with special non-reactive radical reagents, such as nitroxide, used as spin-labels, whose chemical environment can be then inferred from the EPR spectra [86]. Another approach to RNA structure determination is based on binding paramagnetic metal ions to the RNA, which enables to probe metal-ion binding sites. Although NMR and EPR share some similarities, the properties of the electron spin utilized in the second method allows for obtaining constraints of greater distances. Continuous-wave EPR measurements are based on the dipole–dipole coupling and provide the distance restraints of approximately 20 Å, while relaxation enhancement—up to 30–40 Å. Finally, pulsed electron double resonance (PELDOR) spectroscopy, also referred to as double electron-electron resonance (DEER), enables to measure long-range distances (∼80 Å) between nitroxides and it can be used to reveal conformational changes in nucleic acid molecules [86,87]. Moreover, spin labels function as probes sensitive to the nucleotide dynamics. Hence the EPR spectroscopy can be successfully applied for studying RNA interactions with various ligands. EPR-based methods have been used to study structural and dynamic features of RNA, to explore different modes of RNA–ligand interaction, to obtain long-range structural restraints, and to probe metal-ion binding sites [88,89], also in combination with NMR [90].

#### Förster resonance energy transfer

Förster resonance energy transfer (FRET) is a technique that measures non-radiative energy transfer between two light-sensitive molecules (chromophores). In typical applications relevant to structural biology, both the donor and the acceptor chromophores are fluorescent, and the term ‘fluorescence resonance energy transfer’ is often used instead. Intrinsic fluorescence of RNA is relatively low, thus for FRET analysis, the potentially interacting molecules or their parts have to be labeled at specific positions with donor and the acceptor fluorophores. The energy transfer occurs by means of intermolecular long-range dipole–dipole coupling, which is distance dependent [91]. FRET is highly efficient when the emission and absorbance spectra of the fluorophores overlap and if they are positioned within the Förster radius from each other (the distance at which half the excitation energy of the donor is transferred to the acceptor, typically 3–6 nm) [92]. The FRET efficiency can be determined from the acceptor emission intensity, from the photobleaching rates of the donor in the presence and absence of an acceptor, or from the change in the fluorescence lifetime of the donor. Spatial measurements provided by the technique rely upon the spectral properties of fluorophores incorporated to studied molecule and range between 10 and 80 Å [93]. Thus, FRET can be used to identify molecular proximity at distances relevant to macromolecular structures, making it a useful tool to quantitate molecular interactions and conformational changes. In particular, single-molecule FRET (smFRET) has become a technique widely applied in studying intra- and intermolecular interactions of biomacromolecules, especially RNA [91]. The method allows resolving individual dynamics and subpopulations, which is especially useful in the investigation of dynamic processes such as folding or systems existing in multiple conformations, e.g., riboswitches [94]. The advantage over ensemble methods is that instead of averaging the results, smFRET provides information about conformations of individual molecules.

#### Chemical cross-linking

Chemical cross-linking (XL) is a technique for analyzing macromolecular interactions, which has been widely used in studies of proteins and nucleic acids. It involves the formation of chemical bonds between proximal elements of the macromolecule analyzed. One variant of this approach uses photo-activatable reactive chemical groups incorporated into the molecule before the experiment. For studies on RNA structure and interactions, photosensitive nucleotides have been used, such as 4-thiouridine (4SU). Upon irradiation with far-UV (<300 nm) or near-UV (320–365 nm) light, photo-activated compounds generate zero-length cross-links between residues that interact with each other or at least form transient contacts in the folded RNA structure. An alternative is to employ bifunctional cross-linking reagents, such as cisplatin (CPT), nitrogen mustard (NM), chlorambucil (CHB), cyanuric acid (sTT), bikethoxal (BKT), or phenyl-diglyoxal (PDG) that can react with specific nucleophilic groups found on the surface of protein and nucleic acid substrates. These reagents bridge across chemical groups present in the native molecule, if they are positioned within the cross-linking range specified by the size of the cross-linker [95]. As the cross-linking reaction is carried out in solution where molecular structures and interactions are dynamic, XL can provide a snapshot from an ensemble of various interactions and conformations that exist simultaneously. Intramolecular cross-links provide information on spatial proximities within the molecule analyzed, while intermolecular cross-links provide information on contacts between the interacting partners. Information from XL can be used as distance restraints between proximal residues.

The sites of cross-linking can be identified by alkaline hydrolysis, primer extension, PAGE, or RNA sequencing techniques [96]. Cell-based approaches, such as Photoactivatable-Ribonucleoside-Enhanced Crosslinking and Immunoprecipitation (PAR-CLIP), allow for the identification of the cross-linking sites with a single-nucleotide resolution. 4SU incorporation, which is responsible for the cross-link formation, can be mapped by sequencing RNA molecules converted into a cDNA library [97]. Because of the intrinsic character of the chemical probing, in which the formation of a stable covalent modification leaves a unique mass signature, XL has also been coupled with mass spectrometry analysis (MS). The XL-MS approach allows for detecting the cross-linked moieties (oligonucleotides, peptides, and individual residues resulting from the hydrolytic or gas-phase fragmentation of the probed material) that were chemically bound to each other. Conjugates analyzed by XL-MS with bifunctional cross-linkers can be analyzed with Links and MS2Links [98], e.g., through the ms3d.org portal [99]. These programs can handle RNA interactions, as well as DNA, proteins, and any of their hetero-conjugate combinations. XL-MS has been applied for studying RNA–protein interactions, in particular for identifying RNA-binding sites in RNA-binding proteins [100,101]. The application of bifunctional cross-linking reagents has been tested for their ability to provide vital spatial constraints on well-characterized RNAs [95]. See also ‘RNA 3D structure modeling approaches that use local structural features and pairwise distance restraints (including contact information)’ section for applications in modeling.

#### Ligation-based techniques

Ligation-based techniques exemplified by RNA proximity ligation (RPL) are a family of methods for studying interactions within or between RNA molecules by reconnecting the ribonucleotide backbone. Typically, RNA molecules under study are initially fragmented to introduce breaks in the RNA chain and to generate chemically reactive 5′ and 3′ termini. Then, chemical ligation of spatially proximal termini of RNA fragments is induced, followed by sequencing to identify ligation junctions in chimeric reads. Some variants of this approach include PARIS [102], LIGR-seq [102], and SPLASH [103], add an inter- or intramolecular cross-linking step to hold the interacting fragments together, and these methods present different strategies of fragmenting and enriching cross-linked RNA molecules. Structural information obtained from ligation-based techniques is similar to that from XL-based methods.

### Probing methods that generate structural information in the form of local features

Enzymatic or chemical structure probing methods are often used in footprinting experiments, to obtain information about local structural features of ribonucleotide residues, such as solvent accessibility, paired/unpaired state, or rigidity/flexibility. Such 1D features do not indicate which ribonucleotide residues are mutually interacting, and hence they cannot be used to determine the secondary or tertiary structure. However, they can be used to inform computational methods for higher order structure prediction, e.g., to find the best match between experimental results and possible patterns of interactions, especially when correlations between structural features of different residues are considered.

Probing techniques involve enzymatic cleavage and/or chemical modification of the RNA, followed by the detection of modified residues and/or sites of cleavage via reverse-transcribing the modified RNA to cDNA. The cDNA synthesis starts with a primer (bound to a site within the RNA molecule or to a special adapter sequence added at the 3′ end) and the reverse transcriptase halts at cleavage sites as well as may halt at modifications, leading to cDNA truncations (RT-stop). Some techniques employ the ability of the RT to misincorporate non-complementary residues for the modification sites (RT-map). Sequenced cDNA reads are computationally aligned to map the positions affected by probing and to calculate average reactivities for all residues in the RNA under study. Reactivities can be then used to represent the relative strength or prevalence of particular structural features, e.g., as restraints or constraints in various types of computational structure prediction. Primer extension by the reverse transcriptase is problematic for small RNA molecules that may not allow for the stable annealing of short primers and for RNA molecules with very stable secondary structures that may cause the RT to stop regardless of any chemical modifications. In these cases, the implementation of MS analysis allows one to dispense with the cDNA synthesis and to directly characterize the chemical adducts that exhibit a characteristic change in molecular mass [104]. The most common types of probing methods are described in more detail below.

#### Enzymatic probes

Enzymatic probes are nucleases that discriminate between paired and unpaired regions [105]. Nucleases such as S1 and P1 and RNase T2 cleave phosphodiester bonds in unpaired regions of RNA (ssRNAs), in a sequence-independent manner. RNases such as T1, U2, and A are sequence-dependent, and they cleave ssRNAs only after specific nucleotides. Conversely, RNases such as V1 preferentially cleaves phosphodiester bonds in double-stranded regions of RNA (dsRNA). The use of data from ssRNA and/or dsRNA-specific cleavage allows measuring the degree to which each ribonucleotide residue is in a single- or double-stranded conformation, which in turn can be applied to obtain information about the secondary structure [106,107]. It should be emphasized that RNases are relatively bulky reagents and they can cleave RNA only in sites that are exposed to the solvent and provide sufficient space for protein binding to the nucleic acid substrate.

#### Chemical probes

Chemical probes may be used as footprinting reagents to interrogate various structural features, including base pairing, local nucleotide dynamics, and solvent accessibility. Chemical reagents typically used for RNA structure probing can be divided into ones that modify bases and are sequence-specific, and those that act on backbone in a sequence-nonspecific manner.

**Base-specific probes** that modify the Watson–Crick (WC) edge of specific ribonucleotides include dimethyl sulphate (DMS), 1-cyclohexyl-3-(2-morpholinoethyl)carbodiimide metho-*p*-toluene sulfonate (CMCT), and 3-ethoxy-a-ketobutyraldehyde (kethoxal). They can be used to discriminate between bases that have the WC edge involved in pairing (and hence protected from modification) from those that have the WC edge exposed and are therefore more reactive. It should be emphasized that having the WC edge involved in pairing does not necessarily indicate the involvement of a given residue in secondary structure, as the WC edge can be used for the formation of various non-canonical interactions with bases, backbone, and with ligands bound to RNA.

**Light-activated structural examination of RNA (LASER)** technique is used to obtain solvent accessibility information of purine nucleobases in RNA molecules [108]. This technique involves the use of UV light to activate nicotinoyl azide (NAz) molecules into reactive electrophilic aroyl nitrenium ions, which form adducts with solvent-exposed purine nucleobases. This technique can be applied *in vitro* to monitor RNA structural changes, e.g., resulting from ligand binding, and it can also be applied *in vivo* for studying RNA structures and interactions.

**Hydroxyl radical footprinting (HRF)** technique employs sequence-nonspecific cleavage of the nucleic acid backbone in the presence of hydroxyl radicals that are generated by decomposition of hydrogen peroxide into a hydroxyl radical and a hydroxide ion in the presence of Fe(II)-EDTA [109,110]. Regions of the molecule that are buried within the structure or protected by interactions with other molecules are less sensitive to the hydroxyl cleavage than those exposed to the solvent. The effect of the cleavage is quantitated at each nucleotide position, and the resulting reactivity profile can be interpreted in the structural context. Fe(II)-EDTA can be dissolved in solution, providing a comprehensive overview of solvent-accessible compared with solvent-inaccessible residues in the molecule under study. Alternatively, it can be chemically tethered to a specific position to act as a local probe to cleave residues within the range of the tether, often approximately 25 A. The distance between a cleavage site and the probe is proportional to the intensity of the cleavage, which may provide pairwise distance restraints for modeling. Multiplexed hydroxyl radical (•OH) Cleavage Analysis (later renamed MOHCA-gel) utilizes the random incorporation of Fe(II)-EDTA-tagged nucleotide analogs followed by cleavage pattern readout via 2D gel electrophoresis [111].

**Selective 2′-hydroxyl acylation analyzed by primer extension (SHAPE)** [112,113] technique uses a sequence-unbiased reagent, e.g., NMIA [113], 1M7 [113,114], 1M6 [115], BzCN [116], NAI [117], NAI-N_3_ [118], and FAI [117], to form an adduct with the sugar 2′-hydroxyl group of flexible or disordered nucleotides, and discriminates them from residues that are rigid, e.g., those base-paired in helices [109,110,112,113]. There is essentially no relationship between SHAPE reactivities and solvent accessibility, as many ribonucleotide residues with unconstrained 2′-hydroxyl groups that are fully buried, react readily with SHAPE reagents. SHAPE thus probes the local nucleotide flexibility of RNA nucleotides regardless of solvent accessibility [119].

#### High-throughput probing

High-throughput probing involves the application of structure-selective reagents to many RNA molecules at the same time, coupled with high-throughput sequencing. This method allows for the investigation of very large RNA molecules, different mixtures of RNAs (even whole transcriptomes), for analyzing different conformational states of RNAs (e.g., in cotranscriptional folding or in riboswitches), and for detecting correlated changes at different positions in the sequence, which may be indicative of long-range interactions. There are many varieties of high-throughput RNA probing techniques developed to probe RNA structure *in vitro* and/or *in vivo*. In particular, a number of methods were developed that use RT-stop as reactivity detection strategy, which differ in the probing reagents and in the priming strategy (defined or random primer) used to prepare a library for high-throughput sequencing. High-throughput methods for RNA enzymatic probing include PARS with RNase S1 vs RNase V1 [120], FragSeq with RNase P1 [121], PARTE with RNase V1 across different temperatures [122], and dsRNA-seq with RNase One [123]. HRF-seq is a parallel sequencing-based method for probing RNA accessibility with hydroxyl radicals [124]. MOHCA-seq is an adaptation of the MOHCA-gel technique, in which the detection of radical cleavage sites is detected by high-throughput sequencing [125]. Also, many high-throughput variations of the chemical probing technique were developed, which utilize RT-stop and/or RT-map approaches. These techniques have been recently reviewed in detail [126].

**Mutational profiling (MaP)** is a high-throughput chemical probing approach that employs detection of multiple chemical modification positions within a single RNA by the RT-map method. The advantage of the MaP method over other sequencing-based probing methods is that a single RNA molecule with the misincorporated nucleotide is analyzed, which eliminates the multiple ligation steps required during sequencing. The sequencing readout is then processed to obtain information about the reactivity of each nucleotide, which in turn can be used to infer structural features. In SHAPE-MaP, the reactivities are used primarily for discriminating between paired and unpaired regions, and they can also be used for identifying competing and alternative RNA conformations or quantitate any process or function that modulates local RNA dynamics [127,128]. This technique can also be used for probing RNA *in vivo* with 1M7 as a probing reagent [129,130]. RNA interaction groups by MaP (RING-MaP) uses DMS as a modifying reagent and can be used to obtain information about through-space interaction networks that define the tertiary structure and govern sampling of multiple conformations [131].

**Mutate-and-map (M^2^)** is a hybrid method, which combines mutational analysis with chemical mapping to detect interacting residues. It relies on a principle that sequence perturbations at specific sites may release interacting partners that are distant in sequence, leading to detectable changes in their modification by chemical reagents. Thus, the RNA molecule is systematically mutated at each position to generate a series of variants, whose structures are then analyzed by chemical probing. The correlations between mutation sites and the associated spatially distant perturbations in probing patterns are then used to infer pairwise interactions in particular canonical base pairs [132].

### Comparison and combination of experimental techniques

Experimental techniques presented above provide different types of structural information, and their application poses distinct challenges. Many RNAs have proven recalcitrant to structure determination by individual methods, in particular, those aiming at capturing the complete structure at atomic resolution. However, a combination of various experimental and computational methods called a hybrid approach has proven to be a successful way of determining structures for even the most ‘difficult’ RNA targets, overcoming the weaknesses of methods applied separately.

MX and NMR are methods of choice to determine RNA 3D structures at the atomic resolution. Structures based on crystallographic data are often merely snapshots of the molecule that in the native conditions adopts multiple conformations, in contrast with those obtained through NMR provide an ensemble of conformers of the structure of the molecule under study. The shortages of MX and NMR, which include the size limitation of the molecule or complex under study, can be tuned by applying other techniques such as cryo-EM, SAS, and AFM that provide low-resolution information concerning the global shape of the studied targets in the native conditions.

SAS methods deliver low-resolution tertiary structure information of a molecule under physiological conditions, spanning also disordered or flexible regions, as well as facilitate the analysis of molecules’ dynamics, i.e., conformational changes, complex formation, folding, movement of flexible domains, and structural alterations occurring in response to changes exerted by external conditions. SAXS and SANS data processed alone can be only used to build coarse-grained structural models that represent the averaged distribution of atoms in the molecule. SAS methods can also be used in combination with other techniques [133]. One of the recent examples is the use of SAXS together with the microfluidic technology as well as with ensemble modeling with MD to provide a landscape of RNA conformations [57].

Constraints regarding the medium or long-range interactions, or protein binding sites, can be measured by chemical probing and footprinting methods combined with MS, EPR, or FRET, which significantly improve the data interpretation. Moreover, scattering measurements are not limited by the size of the molecule of interest and they supplement the methods such as solution state NMR or cryo-EM, generally restricted to small and large targets, respectively. A combination of methods revealing the overall shape with techniques focussing on local structural information allows for the structure determination of molecules exceeding the limitations of a single approach, exemplified by solving the structure of the 30 kDa HIV-1 RNA Dimerization Signal by combining cryo-EM, NMR, and MD [64].

**Figure 1:**
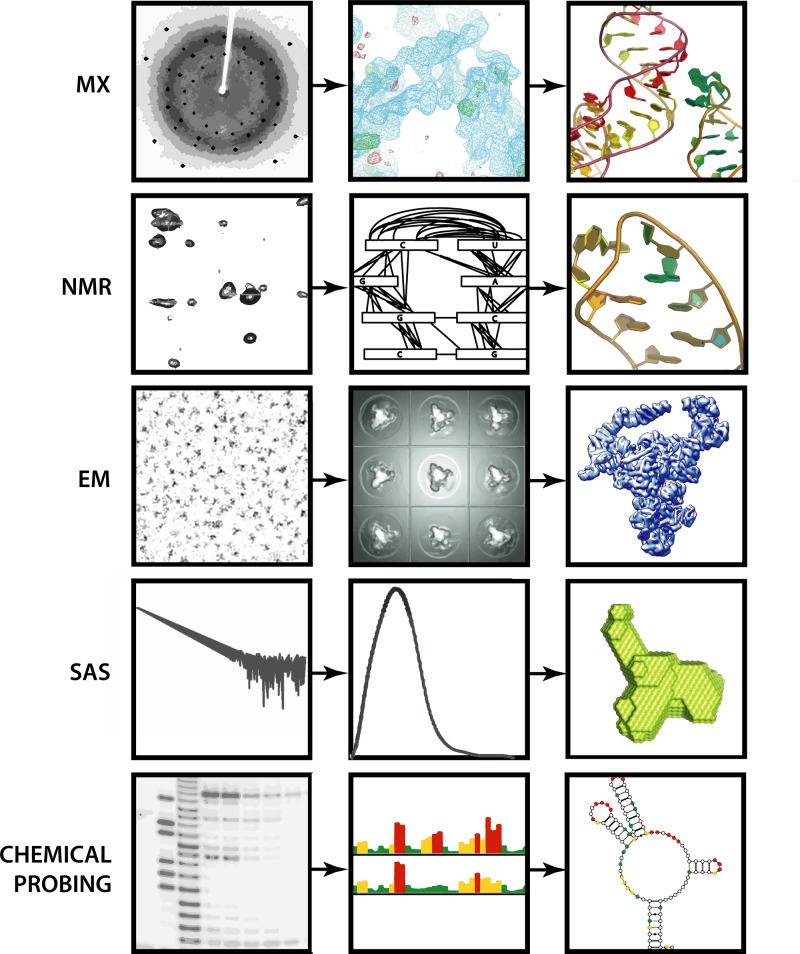
Schematic illustration of selected experimental methods applicable to RNA structure determination: from data acquisition, through data processing, to the generation of an atomic model, 3D shape, or secondary structure representation

Table 1 summarizes the main features of experimental methods applicable to RNA structure determination. Figure 1 illustrates schematically the various stages of data acquisition and processing toward the determination of RNA structures by selected commonly used experimental approaches.

**Table 1:** Experimental methods commonly used for RNA structure determination

Experimental technique	Data type		Main disadvantages and bottlenecks	Number of 3D structures in the PDB		Resolution [Å] of 3D structures in the PDB		RNA length (nt) in 3D structures in the PDB	
	Original	Interpreted		RNA	RNP	RNA	RNP	RNA	RNP
MX	Diffraction patterns	Electron density maps	• Obtaining well-diffracting crystals, especially for flexible or disordered molecules • Phasing in the absence of a related structure	807	1680	0.61–11.5	1.14–11.5	4–2880	2–3396
SAXS/SANS	Electron/neutron scattering patterns	Low-resolution envelope	• Aggregation at high concentration • Heterogeneity of the sample	NA	NA	NA	NA	NA	NA
EM (including cryo-EM)	2D projections of sample	3D density map	• Challenges of working with a liquid sample	29	478	4.5–18.8	2.5–34	12–2904	2–5070
Solution NMR	Free induction decay signal	NMR spectrum/atomic interactions	• Aggregation • Size of the target biomolecule	503	120	NA	NA	5–155	4–101
ssNMR	Free induction decay signal	NMR spectrum/atomic interactions	• Broad line widths and resonance overlap	1	0	NA	NA	26	NA
SHAPE/ Probing methods	Stops/mutations in cDNA reversely transcribed from modified RNA	Reactivity profile	• Reproducibility • Data interpretation in the structural context	NA	NA	NA	NA	NA	NA
FRET	Dampening of energy	Distances between interacting chromophores	• Reduced signal-to-noise ratio associated with acquiring the complete spectrum	1	0	NA	NA	NA	49
Fiber Diffraction	Diffraction patterns	Electron density maps	• Loss of structural information due to cylindrical averaging of diffraction data	0	5	NA	2.9–3.5	NA	3 - 3
Combination of experimental techniques			• Combining data obtained by different techniques	4	1	NA	NA	47–192	58
Total				1345	2284				

## RNA 3D structure modeling methods informed by experimental data

Purely theoretical prediction of RNA structure, which disregards all experimental information, is currently not practical. While it is known that all biophysical processes, including RNA structure formation, ultimately depend on the quantum chemistry, the enormous mathematical complexity of computational quantum mechanics methods limits their application to systems up to a few hundreds of atoms [134,135]. However, RNA molecules alone are often composed of hundreds or thousands of atoms, and the simulation must also take into account solvent molecules. In general, all contemporary RNA structure prediction methods that are capable of making biologically useful predictions rely on experimental information.

Thermodynamic experiments such as the UV optical melting and differential scanning calorimetric measurements were used to determine the folding stability of short RNA strands and to infer parameters for secondary structure formation, which are now commonly used in computational methods (review: [136]). For the development and testing of computational methods for RNA 3D structure modeling, datasets of experimentally determined macromolecular 3D structures are fundamental. In particular, datasets of high-resolution structures determined by MX and NMR, available in the PDB, were used to derive statistical parameters that describe the structural and dynamical features of RNA molecules. These parameters describe the frequency of different molecular features and can be encoded in the form of scoring functions used in computational folding simulations, e.g., to estimate the energy of different conformations, or to assess the models according to their similarity to structures observed previously. For instance, statistical information on torsion angles and sugar puckers can be used as prior knowledge in structure prediction algorithms that rely on conditional probabilities [137,138].

### RNA 3D structure modeling approaches that use atomic coordinates of known structures as the primary source of experimental information

The experimentally determined macromolecular structures have been widely used as a source of ‘spare parts’ in the process of model building, from structures and conformations of chemical groups comprising several atoms, to fragments comprising several residues, to local 3D motifs, and to whole molecules. The most successful programs available for molecular modelers often combine multiple approaches, however for the purpose of this review we define the key modeling approaches separately from the actual implementations.

**Comparative modeling** (also called **homology modeling** or **template-based modeling**) technique is based on the observation that evolutionarily related (homologous) molecules adopt very similar structures, despite divergent mutations accumulated in their sequences [139]. Thus, a ‘template’ molecule can be used as a structural framework, in which the original sequence is ‘mutated’ to become the sequence of the ‘target’ molecule while retaining most of the local and global structural features. The identification of a template structure (one or more) and aligning the target and template sequences (to determine residues that are spatially equivalent) is the first and the most crucial step of this kind of modeling. Thus, the success of comparative modeling is limited by the availability of experimentally solved RNA 3D structures that can serve as templates, and hence this approach is heavily dependent on macromolecular structure databases.

Typically, comparative modeling can produce highly accurate models for target molecules that exhibit high sequence similarity to the templates, indicative of a close evolutionary relationship. Evolutionarily related RNAs with divergent sequences often retain the ancestral tertiary folds, very much like proteins do [140]. However, with the increasing evolutionary distance (and sequence dissimilarity) between the target and the template, the likelihood of obtaining an accurate model decreases, due to the structural divergence and mounting difficulties in the generation of accurate sequence alignments. Technically, it is possible to carry out comparative modeling using templates that are evolutionarily unrelated to the target, but this requires the unrelated template to be structurally very similar to the target. Comparative models based on wrong templates, or on correct templates with wrong alignments, are bound to be wrong.

In the absence of closely related molecules in the database of experimentally determined structures, the detection of templates becomes challenging. This issue is addressed by fold-recognition (FR) methods, which aim at predicting which of the known 3D structures is likely to exhibit a common architecture with the target sequence. FR has been developed for proteins and while it is applicable to RNA, to our knowledge it remains to be implemented in this context. FR can involve sequence similarity searches (typically to identify molecules with a known structure that are evolutionarily related to the target) or threading (finding structures that can accommodate the target sequence in such a way that favorable contacts are formed in 3D) or the combination of these approaches.

Comparative modeling methods often use atomic coordinates of experimentally determined structures in one of two ways. One group of methods, exemplified by ModeRNA [141,142], directly copies atomic coordinates of the structural core from the template, preserving the backbone in regions of continuous alignment, and ‘patches up’ backbone discontinuities caused by insertions and deletions by stitching the template with short fragments from other structures. Whenever the template structure or additional fragments disagree in sequence with the target, bases are replaced by geometry matching. These type of methods do not require any energy calculations, and scoring is used mostly to minimize gaps in the backbone and steric clashes between the inserted fragments and the core. Another group of comparative modeling methods, exemplified by Modeller [143] or MacroMolecular Builder (MMB, previously known as RNABuilder) [144], use target-template alignment to assign correspondences between residues, and then assign spatial restraints (e.g., bond lengths and torsion angles) to the target sequence, based on the values for corresponding residues in the template. The target sequence is then folded to satisfy these restraints. Also, energy minimization is performed with the use of a force-field that ensures the resulting structure is physically realistic. Additionally, geometry constraints can be used based on general statistics from RNA 3D structures to enforce proper stereochemistry. In both types of approaches, secondary structure information can also be used to constrain the selection of fragments or to restrain the helices to achieve proper interactions and geometry.

**Fragment assembly** is another approach for structure prediction, which can use atomic coordinates of experimentally determined structures. As opposed to comparative modeling, it does not require a match of the template sequence to any particular structure, so it is essentially a template-free approach. Instead, it builds the structure *de novo* by using segments extracted from known 3D structures as building blocks. Often, the modeling procedure involves the testing of various combinations of fragments, which are assessed by a scoring function that can be based on statistics, physics, or both. Typically, secondary structure information is used in the form of constraints in the process of fragment selection. Additional spatial restraints may also be used. Both in comparative modeling based on restraints and in fragment assembly, additional information may also be encoded: pairwise distance restraints are relatively straightforward to encode and are enabled by most methods, and some methods (e.g., MMB) also allow 3D shape restraints in the form of density maps. There exist methods for manual (semi-automated) modeling with the use of fragment assembly such as RNA2D3D [145] and Assemble [146,147]. There are a few fully automated methods based on a computational assembly of RNA fragments, such as RNAComposer [148] and Rosetta/FARNA/FARFAR [149,150].

### RNA 3D structure modeling approaches that use local structural features and pairwise distance restraints (including contact information)

***De novo* modeling** is an approach for structure prediction, which by definition does not use templates. ‘*Ab initio*’ modeling methods that rely solely on first principles (i.e., on quantum physics calculations), even with various simplifications, are extremely costly and are incapable of predicting 3D structures for objects as large as typical RNA structures starting from sequence information alone [151]. On the other hand, most *de novo* folding methods enable simulating the RNA structure formation process based on sequence alone or supplemented only with secondary structure information. Therefore they introduce various simplifications and biases to make the prediction process plausible, by trading off accuracy for speed, given limited computing resources. Many of these methods use a coarse-grained representation and simplify or ignore various aspects of RNA structure and dynamics that are not essential for predicting the 3D structure that is as close to the real one, as possible and feasible [134,152]. All successful methods for *de novo* modeling of RNA structure, do utilize prior information on experimentally determined structures, very often in the form of statistics that guide the folding toward conformations that are similar to those observed earlier. It has been shown, e.g., in the series of community-wide RNA Puzzles experiments, that the use of additional information in the form of various additional restraints, contributes greatly to the accuracy of predictions [153–155]. In fact, for large RNAs, it is still virtually impossible to predict 3D structures accurately (with RMSD to the native structure <10 Å) without the use of structural restraints.

Essentially all methods for RNA 3D structure prediction can use restraints or constraints on secondary structure. Many of the *de novo* folding, as well as fragment assembly methods, actually require a secondary structure to be included as one of the elements of the input, so they either take the secondary structure supplied by the user or they predict it using one or more of the external tools. Many programs can also use additional pairwise restraints derived from different types of experiments, beyond the information about the secondary structure. Multidimensional Chemical Mapping (MCM) is a computational workflow for RNA 3D structure modeling that utilizes Rosetta/FARFAR with restraints based on experimental probing with the M^2^ and MOHCA-seq approaches, which provide secondary structure, and tertiary proximity information, respectively [156]. Examples of RNA 3D structure prediction methods that are capable of using secondary structure data as well as other data types, such as restraints, include NAST [157], iFoldRNA-v2 [158], and SimRNA [159].

A few modeling methods specialize in the use of restraints from NMR experiments. The Al-Hashimi group used ^1^H chemical shift data to restrain MD simulation and suggested the prospects of using chemical shift data as restraints in RNA structural modeling as well as in the qualitative evaluation of RNA dynamic ensembles [160]. ^1^H chemical shift data obtained from NMR experiments can also be used in an extension of the Rosetta method, Chemical-shift-ROSETTA for RNA (CS-ROSETTA-RNA), to model non-canonical RNA motifs using [161]. iFoldNMR can use inter-imino-proton distances and atomic inter-base distances associated with base-pairing configuration obtained through ^1^H–^1^H NMR NOESY and NMR _JN_-COSY experiments, respectively [162].

The integration of XL-MS with computational techniques for molecular 3D structure determination has been initially developed for proteins and termed as MS3D [163]. The feasibility of MS3D for RNA structure determination has been demonstrated for the mouse mammary tumor virus (MMTV) ribosome frameshifting pseudoknot [164] and for the HIV-1 Ψ-RNA packaging signal [165]. However, the analysis of RNA–RNA cross-links by XL-MS is difficult and thus far MS3D has not been established as a routine technique for structural studies of RNA.

### RNA 3D structure modeling approaches that use 3D shape information

While the information about molecular shapes is the basis of several methods for macromolecular structure determination, it is rarely used as a restraint by modeling methods that are largely predictive. However, a number of computational methods were developed that blur the line between data-driven structure determination and data-driven predictive modeling. Such methods perform flexible fitting of molecular structures into density maps obtained from EM or MX experiments. They were developed to improve the model building in particular for maps that are of too low resolution for traditional structure determination methods to produce reliable atomic coordinates, e.g., at the resolution > 3.5 Å for MX. This type of modeling usually does not construct the whole structure from scratch and should be typically initiated with a 3D model that is relatively close to the expected final conformation, and only small to medium structural changes are introduced. For instance, the MD Flexible Fitting (MDFF) method incorporates the EM data as an external potential added to the MD force field, allowing the internal features present in the EM map to be used in the fitting process. In this approach, the expected conformational changes must be within the range of the MD method [166]. A similar task can be performed with different sampling algorithms, e.g., in the DEN approach, a Deformable Elastic Network is used to restrain the sampling to prior knowledge of an approximate structure, which reduces overfitting, especially at low resolution. The DEN restraints can also be used to enhance reciprocal space simulated annealing refinement [167]. The Das group has developed an extension of Rosetta called DRRAFTER (*De novo* RNP modeling in Real-space through Assembly of Fragments Together with Electron density in Rosetta), which can be used to build RNA coordinates into cryo-EM maps of RNAs and RNPs [168].

### Hybrid modeling approaches that involve data-driven modeling

Given the scarcity of experimentally determined high-resolution structures of RNA molecules and their complexes and the wide range of heterogeneous biochemical data, computational techniques can be used to integrate existing data, guide structure elucidation, and subsequently determine the mechanisms of action and interactions between components. This approach is commonly called hybrid or integrative modeling. It has been developed and used mainly for protein complexes, but it has also been applied to systems involving RNA. Typically, the result of integrative modeling is not just a single structure, but rather a series of models that are maximally consistent with the input data. Hence, in the case of insufficient data, a user obtains multiple alternative solutions that can be utilized to plan additional experiments. Theoretical models of macromolecular structures can assist in understanding and guiding the identification of important interaction surfaces and, more importantly, can provide a starting point for higher resolution descriptions.

Integrative Modeling Platform (IMP) is an example of a very comprehensive computational toolbox for hybrid modeling, which encompasses various applications for data-driven modeling. It supports the use of multiple types of restraints that are applicable for RNA modeling, including SAXS profiles, EM images and density maps, and sparse NMR data [37]. There are many different sampling protocols implemented. The preparation of the input data for IMP requires advanced knowledge of the system analyzed. IMP has been used to predict macromolecular structures involving RNA, such as ribosomes [169,170]. Another example of a hybrid modeling method is PyRy3D developed in our group (http://genesilico.pl/pyry3d/), which aimed at the relative simplicity of the input while retaining the ability to use various restraints. This method is particularly useful for data-driven modeling of complexes where the users have only limited knowledge about the system analyzed and wish to test alternative hypotheses, e.g., by using restraints that may be ambiguous or mutually incompatible. PyRy3D enables the construction of models for individual molecules as well as for large macromolecular complexes, and it combines the modeling of rigid bodies (e.g., for components with experimentally determined structures) with flexible shapes (e.g., for components with completely unknown structures or for regions of molecules predicted or determined to be disordered). PyRy3D has been used to model RNA and RNA–protein complexes such as the complex between the 2′,5′-oligoadenylate synthetase OAS1 and the 3′-terminal region of the WNV RNA genome [171]. M3 is yet another computational method that incorporates sparse and hybrid experimental data to develop molecular models, which is applicable to RNA [172]. The M3 protocol uses the HADDOCK framework [173], and accepts input in the form of interatomic distances and/or molecular shapes. Modeling with M3 can account for extensive structural rearrangements both at the domain and atomic levels. There also exist other programs and software packages that support integrative modeling, and their utility for RNA 3D structure modeling remains to be verified.

**Figure 2:**
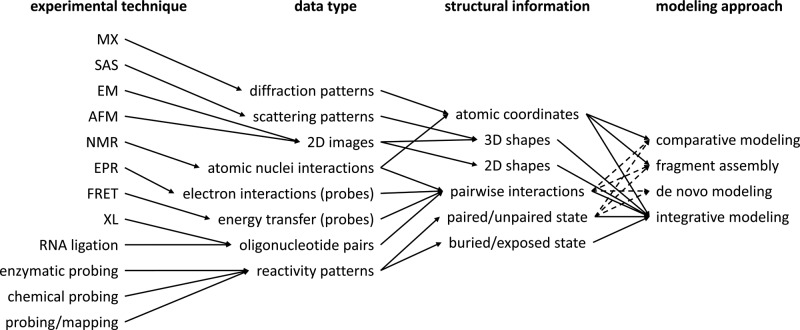
Information flow from experimental techniques to modeling approaches

Table 2 summarizes the main features of modeling approaches, with emphasis on data requirements and limitations of applicability. Figure 2 illustrates the flow of information from experimental measurements to computational modeling of RNA 3D structure.

**Table 2:** Main computational approaches for RNA 3D structure modeling

Modeling technique	Obligatory input data for the target RNA (prior knowledge)	Non-obligatory additional data often used	Main advantages	Main disadvantages
Comparative modeling	• Target RNA sequence • 3D structure of another RNA (at least one) used as a template • Sequence alignment between the target and the template(s)	• Secondary structure	• Computationally inexpensive and hence very fast • Practically no size limitation	• Does not work without the template • Critically depends on the accuracy of the template and the alignment. Models based on a wrong template or on a correct template but with a wrong alignment are bound to be incorrect • Regions that depart from the template structure are modeled poorly
Fragment assembly	• Target RNA sequence • Database of fragments of RNA 3D structures • Assignment of structural fragments to sequence fragments (usually based on predefined secondary structure)	• Secondary structure • Pairwise distance restraints	• Computationally inexpensive and hence very fast • Practically no size limitation	• Usually depends on the accuracy of predicted secondary structure • Complex 3D topologies and pseudoknots are difficult to predict
*De novo* modeling	• Target RNA sequence	• Secondary structure • Pairwise distance restraints • Partial 3D structure	• Often capable of modeling small RNA molecules (<100 nt long) based on sequence data alone, without any additional information	• Computationally expensive and hence relatively slow • Limited to ∼100 nt residues without additional data (longer sequences are modeled very poorly), the limit can be extended by the use of additional data (toward integrative/hybrid modeling)
*Ab initio* modeling	• Target RNA sequence • Detailed chemico-physical conditions of the system (e.g., ion concentration, pH, temperature etc.)		• Enables modeling based on first principles, only with the knowledge of the RNA sequence and environmental conditions, but without any restraints	• Computationally very expensive and hence very slow • Limited to very small systems, which are very well characterized
Integrative/hybrid modeling	• Target RNA sequence • Various types of experimental data (often partial 3D models, secondary structure, shape restraints, pairwise distance restraints)	• Most data types used are non-obligatory, but the modeling procedure is mostly data-driven	• Enables modeling based on the combination of various types of experimental data	• Applicable to large systems • Computationally expensive and hence relatively slow • Critically dependent on the quantity and quality of data

### Assessment of RNA 3D structural models

The ability to discriminate correct models from incorrect models is of great importance for RNA 3D structure prediction. When the experimentally determined structure is available as a reference, a number of computational methods can be used to make *a posteriori* assessment [174] about the accuracy of models. However, in real life cases, the ‘true’ RNA structure is not available, which defines the purpose of RNA 3D structure prediction. Therefore, the challenge is not only to build ‘a model’ or ‘models’ of that RNA structure, but also to assess how similar these models may be to the unknown ‘true’ structure. Generally, *a priori* model assessment attempts to answer questions such as ‘is a given structural model likely to be correct or is it likely to be wrong’, ‘which parts of the model are likely to be correct, and which parts are uncertain’ and ‘for a given set of models, which are likely to be the better than others’.

The most obvious way to assess structural models is to compare them with data that were not used in the process of structure prediction and can be considered independent. In crystallography, for instance the model is assessed with the R-work and R-free values. The R-work measures the agreement between the model and the actual experimental data, by calculating a simulated diffraction pattern from the model. The R-work value can be, however, biased as the model can be refined to better agree with the experimental data. A less biased measure is the R-free, which measures how well the model can predict a test set of reflections (5–10% of the dataset) that were not used during refinement [175]. For other types of experimental data, there exist specialized methods dedicated to scoring features of 3D models. Many RNA 3D structure modeling methods, such as MC-Sym [176], NAST [157], RACER [39,177], and SimRNA [159] can use SAXS raw data as a part of the model scoring procedure, to ‘filter out’ conformations that disagree with the experimental shape measurements. In this approach, the theoretical scattering curve of the generated models is calculated and then compared with the experimental SAXS data. MC-Sym generated models obtained with any method can also be scored/filtered with standalone methods, such as Fast-SAXS-RNA [178]. RS3D is another program that uses SAXS data, together with the secondary structure and tertiary contact information, to score RNA structures obtained in a Monte-Carlo simulation [179]. A general purpose method FILTREST3D can score structural models (including RNA 3D structures and RNP complexes) based on a combination of distance restraints with other factors such as local or global structure or molecule shape, and it implements logical operators to enable sets of alternative restraints [180].

What can be done if no additional experimental data exist? A 3D structural model can be assessed statistically: first, if the modeled structure exhibits physico-chemical and geometrical features that are generally expected from a well-folded molecule and second, if a particular model is similar to other models, predicted independently by other computational methods. Many methods for the theoretical prediction of 3D structural model accuracy based on these principles have been developed for proteins, and they have been evaluated regularly in the CASP experiment [181]. Thus far, quality assessment of RNA 3D structures have lagged behind, and only a few such methods are available, including standalone potentials for scoring 3D structure models [182–184] and internal scoring functions of RNA 3D structure prediction methods (e.g., ROSETTA, SimRNA, and others). We have recently developed a web interface for assessing RNA 3D structure models with a variety of scoring functions (http://genesilico.pl/mqapRNA/, Marcin Magnus and Janusz M. Bujnicki, unpublished) to facilitate the comparison of different approaches.

Results of RNA Puzzles experiments conducted so far indicate that the assessment of RNA 3D structure model quality is a significant bottleneck in RNA structure prediction, as even the best modelers are unable to predict with confidence which of their own models are likely to be better than others [153–155].

**Figure 3:**
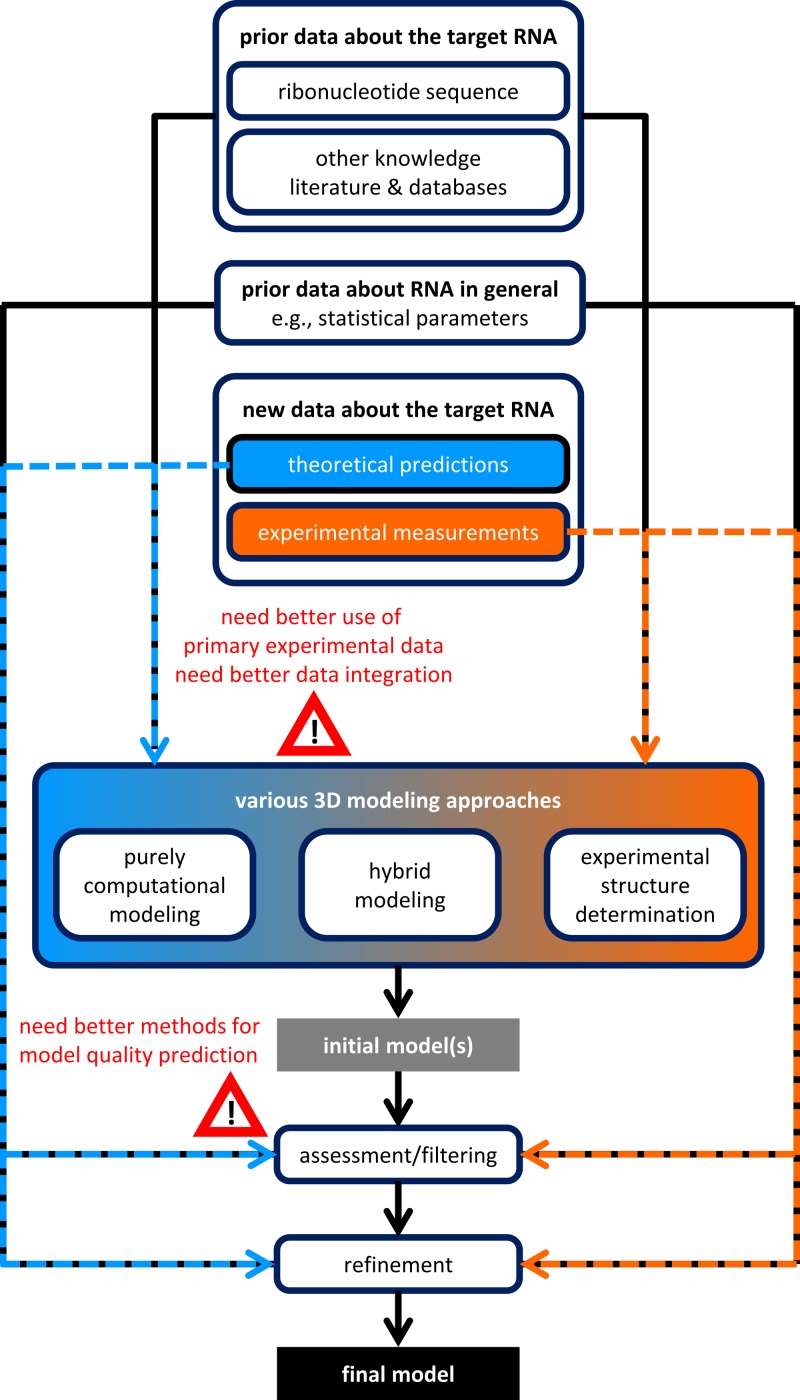
Data workflow in RNA 3D structure determination, with various experimental and theoretical approaches, and combinations thereof

Figure 3 illustrates a general workflow of macromolecular modeling applicable to RNA, highlighting the use of experimental data. Major methodological bottlenecks are indicated to suggest areas for future improvement.

## Which types of experimental data are available, but are not yet commonly used by modeling methods? How can they be implemented?

Large-scale structural studies of RNA gained momentum much later than efforts to systematically determine protein structures such as ‘structural genomics’. In the last decade, many of the strategies used to model 3D structures of proteins have been adapted for RNA structural modeling [185]. Nonetheless, there are some types of experimental data used in protein modeling that have not yet been implemented for RNA 3D structure modeling.

In MX, experimental phasing methods are required for *de novo* structure determination in order to solve the so-called phase problem. Both anomalous dispersion and isomorphous replacement approaches require the incorporation of heavy atoms within the crystals, and the structure determination process relies on the determination of their location (substructure determination) through Patterson or direct methods. The diffraction data collected in the presence of heavy atoms is usually of lower resolution than the data collected from unmodified crystals, and thus the electron density maps obtained after phasing suffer from a lack of features that can make the manual model building very challenging or even impossible. The situation can drastically improve if native data at better resolution and a partial model are available for phase extension. Obtaining the latter could be used by modeling programs that, taking advantage of low-resolution electron density maps, would be able to place helices, known 3D structural motifs or other distinguishable structural features to yield a partial model for phase extension and subsequent all-atom model building.

As mentioned earlier, electron density maps from MX and EM have been used as restraints for modeling of RNA and RNP 3D structures. However, this has been largely limited to data at medium resolution, where individual residues can be distinguished from each other. On the other hand, the EM technique can generate low (e.g., >10 Å) and very low (>>10 Å) resolution maps for macromolecules of unknown structure, which could be used as potentially useful restraints by the modeling methods. In principle, the restriction of the conformational space by any shape restraints is very helpful for modeling, as it saves the time that could be otherwise wasted on analyzing completely wrong molecular architectures. Instead, conformational sampling can be focussed on testing models that are more likely to be similar to the true structure.

2D class averages calculated from cryo-EM micrographs are used in predicting 3D structures of protein assemblies [186,187]. This approach bypasses the necessity of single-particle reconstruction step to obtain a complete 3D density map. Instead, 2D projections of computationally generated 3D models are generated and compared with experimentally obtained 2D projections. This approach has not yet been commonly used for RNA molecules and could be incorporated in RNA 3D structure modeling programs.

Another type of data that could be very useful for RNA modeling is offered by AFM. With the recent advances in this technique, RNA molecules can be visualized to reveal features such as double- and single-stranded regions, and their interactions [66,188]. AFM can be particularly useful for structural studies of relatively big RNA molecules with multiple secondary structure elements that can be arranged on a surface while preserving the geometry of local motifs [67]. Importantly, AFM, as a single-molecule technique can reveal alternative conformations. In principle, AFM images could be used to encode shape restraints in a similar way to 2D class averages from cryo-EM experiments.

Restraints from XL-MS and MS-MS experiments are used in modeling of proteins and their complexes with other macromolecules including RNA–protein complexes [100,189,190]. There are multiple approaches available to access the quality of the theoretically predicted 3D model of proteins using XL-MS data [191–193]. However, despite its success in protein structure analysis and determination, the use of XL-MS has been limited in RNA structural studies, mostly due to the difficulties associated with experimental data collection and analysis. RNA sequences are composed of only four basic building blocks, therefore the unambiguous determination of short oligonucleotides in cross-linking products is much more difficult than for peptides. However, some methods for computational modeling of RNA 3D structure are capable of handling ambiguous restraints or could be modified to enable such functionality. Thus, even if the XL-MS experiment yields a highly unambiguous dataset in which every cross-link can be assigned to a whole list of pairs of oligonucleotide fragments from a given sequence, this could be used as an element of the scoring function, e.g., to eliminate models that are largely incompatible with any of the XL-MS assignments.

Last, but not the least, computational methods for RNA 3D structure prediction could be improved in the way how the footprinting data are used. Reactivity profiles, e.g., from SHAPE and related experiments, are usually first used as an input for specialized secondary structure prediction methods, and only then the predicted secondary structures are used as constraints or restraints in the RNA 3D structure modeling process. However, for some RNA modeling methods, it should be possible, and potentially advantageous, to use the probing data directly at the stage of model building. For example, probing data that indicate the likelihood of individual residues to be paired compared with unpaired could be used as an element of the scoring function without the necessity to specify the binding partners. This could be particularly advantageous for predicting complex RNA structures with pseudoknots that are notoriously difficult to infer for secondary structure prediction tools. On the other hand, modeling tools such as our own program SimRNA, which make and break base pairs and entire secondary structures as a part of the conformational sampling process, do not require any special treatment for pseudoknots, and could be guided to maximize the agreement of the 3D model directly with the probing pattern [159].

Ultimately, we propose that methods for RNA 3D structure modeling that enable the use of experimental data as restraints could be significantly improved. First, there exists a number of experimental techniques that are incapable of unambiguous structure determination, but they generate data that can be potentially used as restraints in molecular modeling. Many of these data are low-resolution and ambiguous, hence modeling methods would have to be adapted to treat them accordingly, and use appropriate statistical frameworks to allow selection amongst multiple alternatives and to tolerate a relatively high level of restraints that could be completely wrong. Second, modeling methods often use highly processed (interpreted) experimental data, which may be misleading. For example, MX and EM data are usually used at the level of 3D density maps and not at the level of diffraction patterns or 2D images. Likewise, footprinting data are not used directly, but in the form of secondary structure predictions. Therefore, we suggest that significant advancement in 3D structure prediction could be made by adapting the existing methods to use experimental data at the possibly early stage of processing, to avoid unnecessary biases. This may require the development of novel ways of treating the experimental data in the prediction process, and as a result, would bring the theoretical approaches closer to what kind of information is actually recorded in the experiments. Such a direction of development may also prompt the theoreticians to collaborate with experimentalists more closely. Computational approaches that deal with the original data may better capture details of biological processes studied experimentally, and could potentially lead to discoveries of new biological phenomena.

## Abbreviations

AFM: atomic force microscopy
DEN: deformable elastic network
DMS: dimethyl sulphate
EM: electron microscopy
EPR: electron paramagnetic resonance
FR: fold-recognition
FRET: Förster resonance energy transfer
HRF: hydroxyl radical footprinting
IMP: Integrative Modeling Platform
MaP: mutational profiling
MD: molecular dynamics
MMB: MacroMolecular Builder
MR: molecular replacement
MS: mass spectrometry
MX: macromolecular X-ray crystallography
M2: mutate-and-map
NMR: nuclear magnetic resonance
PDB: Protein Data Bank
SAS: small angle scattering
SANS: small angle neutron scattering
SAXS: small angle X-ray scattering
SHAPE: selective 2′-hydroxyl acylation analyzed by primer extension
smFRET: single-molecule FRET
ssNMR: solid-state NMR
XL: cross-linking
XL-MS: cross-linking MS
WC: Watson–Crick
4SU: 4-thiouridine
